# The identification of an integral membrane, cytochrome *c* urate oxidase completes the catalytic repertoire of a therapeutic enzyme

**DOI:** 10.1038/srep13798

**Published:** 2015-09-08

**Authors:** Nicola Doniselli, Enrico Monzeglio, Alessandro Dal Palù, Angelo Merli, Riccardo Percudani

**Affiliations:** 1 Department of Life Sciences, University of Parma, Italy; 2 Department of Mathematics & Computer Science, University of Parma, Italy

## Abstract

In living organisms, the conversion of urate into allantoin requires three consecutive enzymes. The pathway was lost in hominid, predisposing humans to hyperuricemia and gout. Among other species, the genomic distribution of the two last enzymes of the pathway is wider than that of urate oxidase (Uox), suggesting the presence of unknown genes encoding Uox. Here we combine gene network analysis with association rule learning to identify the missing urate oxidase. In contrast with the known soluble Uox, the identified gene (*puuD*) encodes a membrane protein with a C-terminal cytochrome *c*. The 8-helix transmembrane domain corresponds to DUF989, a family without similarity to known proteins. Gene deletion in a PuuD-encoding organism (*Agrobacterium fabrum*) abolished urate degradation capacity; the phenotype was fully restored by complementation with a cytosolic Uox from zebrafish. Consistent with H_2_O_2_ production by zfUox, urate oxidation in the complemented strain caused a four-fold increase of catalase. No increase was observed in the wild-type, suggesting that urate oxidation by PuuD proceeds through cytochrome *c*-mediated electron transfer. These findings identify a missing link in purine catabolism, assign a biochemical activity to a domain of unknown function (DUF989), and complete the catalytic repertoire of an enzyme useful for human therapy.

As a drug used to treat hyperuricemia associated with gout, tumor lysis syndrome, and the Lesch-Nyhan disease, urate oxidase (Uox, also called uricase) is an enzyme of considerable biomedical interest^1^,^2^,^3^. The intravenous administration of urate oxidase is a particular example of enzyme-replacement therapy, in which the enzyme is not just missing in the individual but in the entire species, resulting from progressive mutations of the *uox* coding sequence in hominid ancestors^4^. In consequence of this evolutionary inactivation, urate is the end product of purine catabolism in human and apes, whereas in other mammals the end product is the more soluble allantoin.

In Bacteria, Archaea, and Eukaryotes, where present, the oxidative conversion of urate into allantoin proceeds through a three-step enzymatic pathway^5^. In the first step, uric acid (urate at neutral pH) is converted to 5-hydroxyisourate (HIU) in an oxygen-dependent reaction^6^. In the second step, HIU is hydrolysed to 2-oxo-4-hydroxy-4-carboxy-5-ureidoimidazoline (OHCU), which is decarboxylated to give dextrorotatory allantoin in the third step (Fig. 1a). As HIU and OHCU are metastable compounds with a half-life of 7.2 and 9.6 minutes at physiological conditions, racemic allantoin is obtained *in vitro* as a final product of the Uox reaction. However, in nature the presence of Uox is almost invariably associated with both HIU hydrolase (Urah) and OHCU decarboxylase (Urad). Besides affecting the reaction stereochemistry^7^, the presence of these enzymes appears to be important for the rapid elimination of the metastable intermediates of urate oxidation^8^.

The functional coupling between Uox, Urah, and Urad is reflected by the evolutionary link of the corresponding genes, which are usually present or absent together in a given genome. This link has been key to the identification of *urah* and *urad* as genes associated with *uox*^5^; in prokaryotes, however, cases were also noticed in which both *urah* and *urad* were present in the absence of *uox* genes. This observation was followed in recent years by the identification of alternative urate oxidases—non homologous enzymes involved in the same reaction step. The *uox* gene encodes the first identified urate oxidase^9^, a cofactor-less enzyme with a T-fold domain^10^ that is found in all tree domains of life. The existence of an alternative urate oxidase gene, named *hpxO* (hypoxanthine-xanthine utilization O), has been demonstrated in *Klebsiella* spp. possessing *urah* and *urad* but not *uox*^11^,^12^. The HpxO enzyme has been biochemically and structurally characterized as a flavoprotein belonging to a large family of hydroxylases^13^,^14^. An isofunctional non homologous urate oxidase has been identified in *Xanthomonas* spp. and named HpyO^15^; although HpyO belongs to a distinct family of hydroxylases, similarly to HpxO, it uses NAD(P)H as a cosubstrate and FAD as a cofactor^15^.

From a mechanistic standpoint, Uox and HpxO/HpyO represent distinct solutions to the same biochemical problem. Each of these solutions has advantages and disadvantages. Uox does not depend on cofactors for activity, but its two-electron reduction of dioxygen generates H_2_O_2_. This potent oxidant is readily detoxified by peroxisomes, explaining the organellar localization of the pathway in eukaryotes^16^,^17^,^18^. However, the formation of hydrogen peroxide by the Uox reaction can pose problems with organisms lacking peroxisomes, and is a matter of concern for the therapeutic administration of the enzyme^19^. On the other hand, the four-electron dioxygen reduction catalysed by HpxO/HpyO generates H_2_O, but requires a labile cofactor and a cosubstrate that is consumed in the reaction (Fig. 1a).

Surprisingly, the enzymatic variety above described does not fully account for urate oxidation in living organisms. It has been pointed out that homologs of *uox*, *hpxO*, or *hpyO* genes are not identified in completely sequenced organisms known to perform urate oxidation^15^, and our preliminary search identified numerous species (mostly proteobacteria) possessing *urah* and *urad* but not recognizable uricase genes. As Urah and Urad act on metastable products of urate oxidation, the presence of the corresponding genes could be justified only by the presence of a gene capable of urate oxidation. However, no candidate oxidoreductases can be identified by homology among the genes associated with purine catabolism. To identify this missing gene and possibly a different urate oxidation mechanism, we used here gene network analysis and a data mining technique able to discover association rules between items of an item set^20^,^21^. The application of this technique identified with high confidence genes belonging to COG3748 as the missing urate oxidase. In striking contrast with the known genes involved in urate oxidation, the candidate gene (*puuD*) was found to encode an integral membrane domain (DUF989) fused with a cytochrome *c*. Experiments with deleted and complemented bacterial strains provided evidence for the functional assignment and revealed a novel mechanistic solution for urate oxidation.

## Results

### Identification of COG3748 as urate oxidase

**Figure 1:**
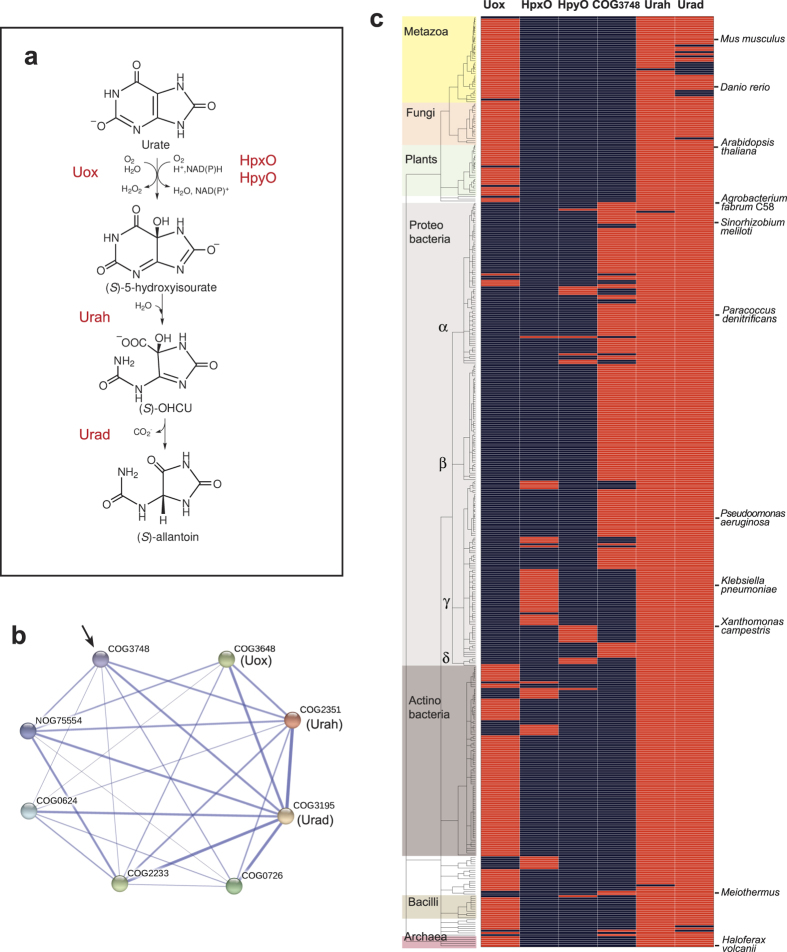
Identification of COG3748 as urate oxidase. (**a**) Pathway for the conversion of urate into allantoin. (**b**) String association network obtained with COG3648, COG2351, and COG3195. Nodes represent gene families according to the COG classification. Edges represent predicted functional associations; stronger associations are shown as thicker lines. The node identified as a candidate urate oxidase (COG3748) is indicated with an arrow. (**c**) Map of urate oxidation capacity in complete genomes. The tree represents 431 distinct species possessing either the *uox*, *hpxO*, *hpyO*, or COG3748 genes or both the *urah* and *urad* genes. The presence (red) or the absence (blue) of genes in complete genomes is shown alongside the organism tree. Main taxonomic groups and organisms discussed in the text are indicated.

To find candidate genes encoding enzymes with urate oxidase activity we interrogated a database of predicted gene/protein associations (String)^22^ using as input Cluster of Orthologous Groups (COGs)^23^ corresponding to the Uox, Urah, and Urad proteins involved in the enzymatic pathway (Fig. 1a,b). Among the numerous associated COGs reported, the COG3748 ‘predicted membrane protein’ ranked in fifth position in the String list (Supplementary Fig. S1) was considered a possible candidate as a domain with unknown function exhibiting the expected relation for a protein with urate oxidase activity, that is a connection with Urah and Urad and not with Uox in the association network (Fig. 1b). Indeed, genes involved in consecutive reactions of a pathway are expected to be correlated (e.g. they are found in the same genomes or in the same operons), while no correlation or anti-correlation is expected for different genes involved in the same reaction. Visual inspection of various operons containing COG3748 genes confirmed the presence of other genes involved in purine degradation and the absence of known genes encoding urate oxidase (Supplementary Fig. S2).

To evaluate in detail the association between COG3748 and the other genes of urate oxidation we examined gene distributions in 1689 complete genomes from different species. Using a semi-automated method for gene classification we found 431 species possessing both the *urah* and *urad* genes or either COG3748 or a known urate oxidase gene (*uox*, *hpxO*, *hpyO*). This distribution clearly shows that the different genes involved in the first step of urate oxidation occur alternatively in different species (Fig. 1c). The cofactor-independent urate oxidase (Uox) occurs in eukaryotes, in some bacteria (particularly Actinomycetes), and in some archaea. Alternative oxidases (HpxO and HpyO) occur in a limited number of bacteria, while COG3748 occurs in several bacterial species (mostly proteobacteria) possessing both *urah* and *urad* but not known urate oxidase genes. Among these species are organisms (e.g. *Paracoccus denitrificans*, *Pseudomonas aerugino*sa) that were previously reported to be capable of oxidative urate degradation^24^,^25^.

Bioinformatics methods make it possible to quantitatively measure correlated^26^ or anti-correlated^27^ gene distributions. Such methods were devised for pairwise associations. The generalization introduced with the analysis of logic implications^28^, however, allows one to evaluate comparisons involving any number of gene families. The gene distribution of Fig. 1c shows that the relations among urate oxidation genes are not well described by pairwise comparisons. For instance, COG3748 is positively correlated with the presence of Urad (Urad implies COG3748 ) and negatively correlated with Uox (not Uox implies COG3748). Although significant, these “association rules”^20^,^21^ have low confidence (Table 1), reflecting the fact that in many cases *urad* is present in the absence of COG3748, and the absence of *uox* does not imply the presence of COG3748 (see Fig. 1c and Supplementary Fig. S3). When more genes were included in the comparisons, association rules with increasing confidence and significance were obtained (Table 1). Interestingly, these associations are consistent with the supposed role of COG3748, and the best association rule obtained (93% confidence; P = 1 × 10^−196^) was exactly that expected *a priori* by biochemical reasoning: that is, the presence of both Urah and Urad in the absence of known urate oxidases implies the presence of COG3748.

Based on the above analysis, we predicted a urate oxidase activity for COG3748. The presence of an integral membrane domain in these proteins (confirmed by sequence analysis, see below) was initially surprising, as the known urate oxidases are soluble proteins. However, early experiments reported that the urate oxidase activity of some organisms, including *P. denitrificans* and *P. aeruginosa*, was lost in the soluble fraction after sonication^24^,^25^, consistent with a membrane-bound enzyme.

### Urate degradation without soluble uricase activity in organisms encoding COG3748 genes

**Figure 2:**
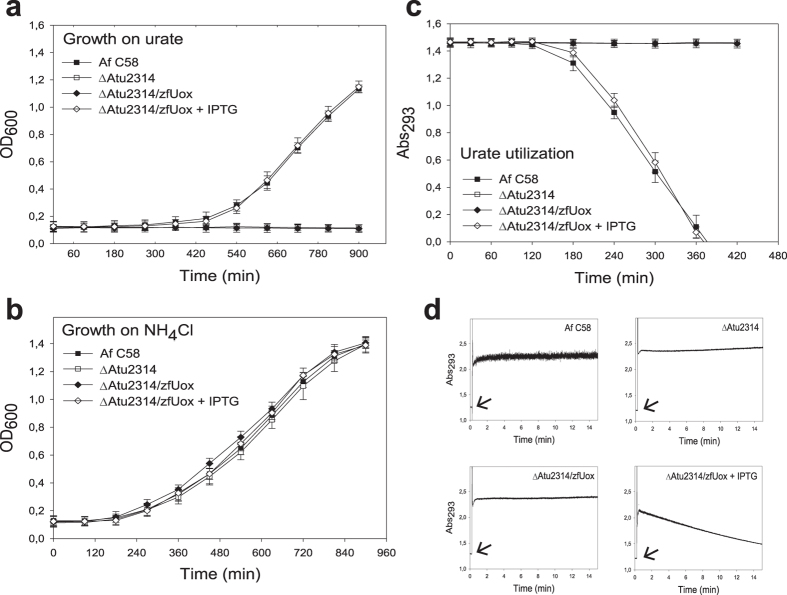
Experimental evidence for the urate oxidase activity of Atu2314 (COG3748). Growth curves of wild-type and engineered *A. fabrum* C58 strains in M9 minimal medium supplemented with (**a**) urate or (**b**) ammonia as nitrogen source. (**c**) Urate utilization by concentrated cell cultures. Error bars represent standard deviations obtained from three independent experiments. (**d**) Uox activity of 250 μg of cell-free extracts as monitored by the decrease in absorbance at 293 nm; extracts were added (arrows) to 0.1 ml solutions containing 0.11 mM urate.

For the anticipated difficulties in the recombinant expression and purification of the integral membrane COG3748 proteins, we sought to validate the bioinformatics prediction using a genetic approach. *Agrobacterium fabrum* C58 (previously known as *A. tumefaciens* C58) was selected as completely sequenced organism^29^ for which well-developed genetic tools are available^30^,^31^. There is no information about urate utilization by *A. fabrum*, although the presence of a COG3748 gene (Atu2314, verified by sequencing of our laboratory strain), *urah* and *urad* genes (see Fig. 1c), as well as other downstream genes of the catabolic pathway^32^, suggest that *A. fabrum* is able to use urate as a nitrogen source. While in animals purine degradation is used to eliminate excess nitrogen, in plant, fungi, and prokaryotes this pathway is important for the recovery of mineral nitrogen from endogenous or imported purines^33^,^34^. Accordingly, we observed growth of *A. fabrum* in a minimal medium supplemented with urate as the nitrogen source (Fig. 2a). With respect to the growth observed in ammonia (Fig. 2b), a longer lag was observed with urate, suggesting that in this bacterium the urate utilization system is inducible. When urate consumption was measured in highly-concentrated cell cultures, the rapid disappearance of the urate peak at 293 nm was observed after a lag of about 120 minutes (Fig. 2c). This lag was not observed with pre-induced cultures (Supplementary Fig. S4). However, no soluble urate oxidase activity could be measured using protein extracts of urate-induced cells (Fig. 2d).

### Expression of a soluble urate oxidase (zfUox) complements the phenotype of Atu2314 (COG3748) deletion mutant

Using a gene replacement vector^30^, we generated a deletion mutant of the Atu2314 gene in *A. fabrum* C58. No differences in the growth on ammonia were observed in the deleted strain (Fig. 2b). By contrast, growth on urate was completely abolished (Fig. 2a), and no urate consumption was observed in the deleted strain (Fig. 2c).

The *uox* coding sequence from the vertebrate *Danio rerio* (zebrafish) was cloned in a IPTG-inducible expression vector^31^ and used to transform the recipient ΔAtu2314 strain. No differences in the growth on ammonia were observed in the deleted and complemented strains in the presence or in the absence of IPTG (Fig. 2a). However, growth on urate was fully restored in the complemented strain in the presence of the IPTG inducer (Fig. 2b). Similarly, urate consumption was observed in ΔAtu2314/zfUox only in the presence of the inducer (Fig. 2c). Interestingly, the growth and urate consumption curves in the wild-type and complemented strains are superimposable, suggesting that in both strains the limiting step is urate uptake of from the medium (Fig. 2a,c). As expected, a soluble urate oxidase activity was measured in protein extracts of the complemented strain grown in the presence of the IPTG inducer (Fig. 2d). By comparison with the activity of the purified zfUox protein (Fig. S5), we estimate the expression of ~1 μg zfUox per 10^6^ cells in the complemented strain, corresponding to an activity of ~3 nmol urate min^−1^. This intracellular activity is ten-fold higher than the activity measured in whole cells, consistent with the notion that urate import in the cytosol is a rate-limiting step in our system.

Based on bioinformatics and biochemical evidence, we conclude that Atu2314 and homologous genes belonging to COG3748 are responsible for urate oxidation in organisms lacking known urate oxidase. As a gene responsible for urate oxidation has been previously approximately mapped on the chromosome of one of such organisms (*P. aeruginosa*) and named *puuD* (mnemonic for purine utilization D)^35^, we adopted this name for the urate oxidase identified here at the molecular level.

### PuuD urate oxidase is an integral membrane protein with a cytochrome *c* domain

Pfam analysis^36^ of the *A. fabrum* PuuD and homologous proteins revealed a bi-domain organization with an N-terminal (aa 3–300) integral membrane domain (DUF898; E = 6 × 10^−135^) and a C-terminal (aa 330–405) cytochrome *c* domain (Cyt_c; E = 3 × 10^−8^). This domain organization was confirmed by the more sensitive HHpred analysis^37^, although this search identified a more significant similarity with another *c*-type cytochrome domain in Pfam (Haem_bd; E = 6 × 10^−13^). Besides the significant similarity to Hidden Markov Model (HMM) descriptions of cytochrome *c*, the presence of this domain is supported by the strict conservation of the canonical CXXCH motif in all PuuD sequences.

**Figure 3:**
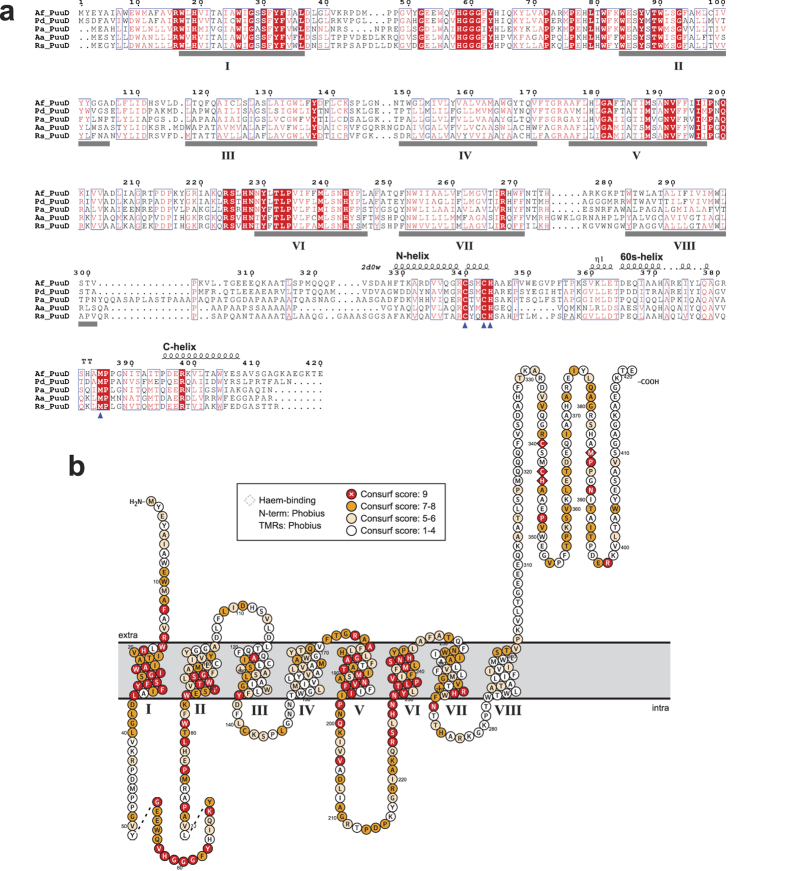
Sequence and structure organization of PuuD proteins. (**a**) Multiple alignment of representative PuuD sequences from *Agrobacterium fabrum* (Af), *Paracoccus denitrificans* (Pd), *Pseudomonas aeruginosa* (Pa), *Acidovorax avenae* (Aa), and *Ralstonia solanacearum* (Rs). Accession numbers are reported in Supplementary Table S1. Colour shading of conserved residues is based on the larger alignment shown in Supplementary Fig. S7. The identified transmembrane helices in AfPuuD are indicated by gray bars and numbered with roman numerals. Blue arrowheads indicate the residues supposedly involved in heme coordination. The secondary structure elements of the cytochrome *c* domain are depicted above the alignment based on the comparison with the PDB structure 2d0w; helices are named according to the mitochondrial cytochrome *c* notation. (**b**) Membrane topology of AfPuuD. Residues are colored according to the Consurf analysis^60^ of the PuuD multiple alignment of Supplementary Fig. S7. Topology plots were obtained with the Protter program^58^.

Sequences representative of the phylogenetic diversity of the PuuD family (Supplementary Fig. S6), where selected to analyse the sequence and structure conservation. The multiple alignment (Fig. 3a and Supplementary Fig. S7) shows high conservation in the N-terminal DUF989 domain, lower conservation in the Cyt_c domain and high variability in the linker region. In the N-terminal domain alignment, eight ungapped hydrophobic blocks are observed. Accordingly, the predictors of transmembrane (TM) domains identified the presence of eight strong TM helices. Consistent results were obtained using single sequences or multiple alignments (Supplementary Fig. S8a,b) and different prediction methods (Supplementary Fig. S8c). Similarly, consistent predictions were obtained for a N_out_-C_out_ membrane topology. With this organization (Fig. 3b), the longer extra-membrane loops (specifically the loops connecting helix I to helix II and helix V to VI) are located in the cytoplasm, whereas the Cyt_c domain is located outside the plasma membrane. Cleavable signal peptides are not identified in PuuD sequences, so in these proteins helix I is assumed to function as a signal anchor for the targeting to the plasma membrane. Within the DUF989 domain, the stronger conservation is observed in helices I, II, V, VI and in the cytosolic loops I-II and V-VI. Within the Cyt_c domain, conservation pertains especially to the residues that interact with the heme cofactor. Invariant residues in all PuuD sequences are two cysteines of the CXXCH motif (Cys340 and Cys343) that covalently bind the vinyl side-chains of heme through thioether bonds plus the histidine (His344) that provides the fifth heme iron ligand. Also, invariant is a methionine residue (Met385) located about 40 residues further on towards the C-terminus, which in *c*-type cytochromes provides the sixth iron ligand.

A search with the PuuD C-terminal domain in the Swissmodel server identified more than 50 distinct cytochrome proteins suitable as templates for 3D homology model reconstruction. The best model was obtained using as template the crystal structure of cytochrome c_L_ from *Hyphomicrobium denitrificans* (PDB 2D0W), a soluble cytochrome of the periplasmic space acting as the physiological electron acceptor of the methanol dehydrogenase quinoprotein through transient protein-protein interaction^38^. Only some parts of the structure can be modelled with high confidence, reflecting the low local similarity (22%) between the template and target sequences (Supplementary Fig. S9a). The positions of residues Cys340, Cys343, His344, and Met385 in the structural model is that expected for heme coordination (Supplementary Fig. S9b), while another invariant residue in the PuuD alignment, Arg398, is directed towards the protein surface and could be involved in protein-protein interactions. Electrostatic bonds are typically involved in the interaction of *c*-type cytochromes with partner redox proteins^39^.

### Role of cytochrome *c* in PuuD urate oxidation

**Figure 4:**
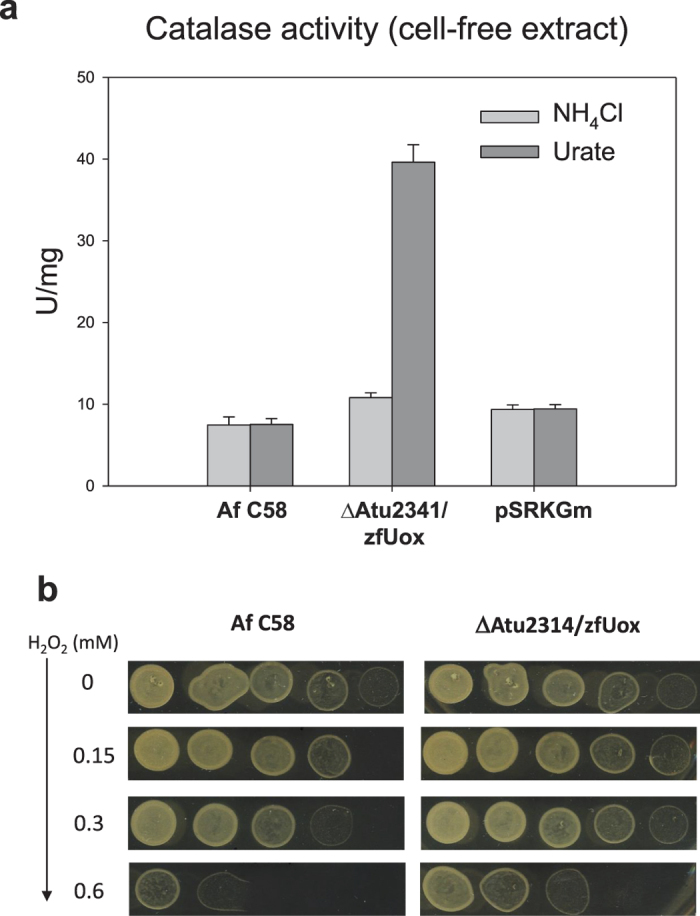
Catalase activity of PuuD and zfUox encoding strains grown on ammonia or urate. (**a**) Catalase activity (U/mg) of free-cell extracts obtained by sonication of urate- and ammonia-grown cells collected during the exponential growth phase; pSRKGm designates Af C58 cells transformed with the empty vector. Error bars represent standard deviation obtained from three independent experiments. (**b**) Urate-grown strains plated on LB agar supplemented with increasing concentrations of hydrogen peroxide. Cells were collected during exponential growth phase, diluted at OD600 = 0.1 and spotted at progressive dilutions (1, 1:5, 1:25, 1:125, 1:625). Scanned plate images were adjusted with −40% luminosity and +40% contrast.

As a cofactor-free oxidase, the zfUox protein used in our experiments to complement the activity of PuuD deletion mutant transfers two electrons to oxygen and generates hydrogen peroxide as a by-product of the reaction. On the other hand, the structure of the PuuD protein and the presence of an electron transfer cytochrome *c* domain suggest a completely different mechanism of urate oxidation. When the catalase activity was measured in protein extracts of ΔpuuD/zfUox cells, a four-fold increase of the catalase activity (p < 0.001) was observed for cells grown in urate with respect to cells grown in ammonia (Fig. 4a). Conversely, no differences in the catalase activity were observed in the parental strain in the two growth conditions. Also, no differences were observed when the parental strain was transformed with the empty vector (Fig. 4a). These results can be explained with production of H_2_O_2_ in the urate oxidation reaction catalysed by zfUox but not in the reaction catalysed by PuuD. The slight increase in the catalase activity observed in the complemented strain grown in ammonia with respect to the other strains (p < 0.1) could be explained by the degradation of endogenously produced urate. Consistent with induction of catalase by the Uox activity, we found that urate-grown ΔpuuD/zfOux strain was more resistant than the wild-type to oxidative stress, as observed by plating cells on agar medium supplemented with increasing concentrations of H_2_O_2_ (Fig. 4b).

Finally, given the presence of a cytochrome *c* domain in the PuuD protein we observed the effect of an inhibitor of cellular respiration (azide) on urate degradation. Azide inhibited urate utilization by *A. fabrum* C58 in a dose-dependent manner, with a 50% inhibition obtained with about 0.2 mM azide. However, a similar effect was observed in the ΔpuuD/zfOux mutant (Supplementary Fig. S10). As the purified zfOux protein was inhibited by azide at much higher concentrations *in vitro* (IC_50_ ~20 mM), we concluded that the inhibition observed *in vivo* was due to a general effect on cellular metabolism rather than a specific effect on the enzymes of urate oxidation.

## Discussion

**Figure 5:**
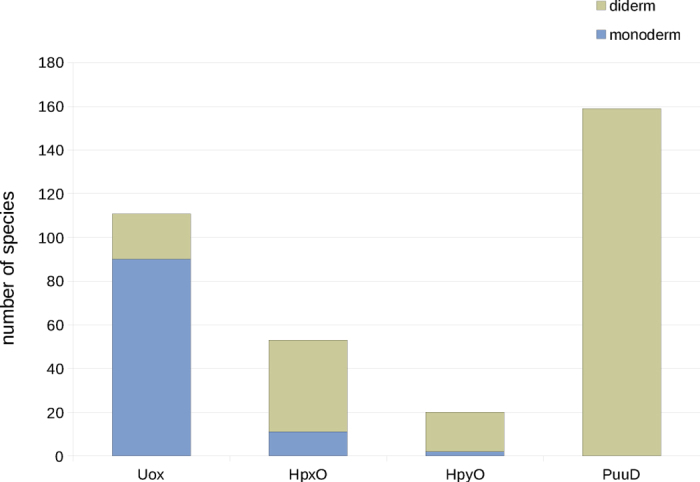
Distribution of urate oxidation genes in monoderm and diderm prokaryotes. The occurrence of the different genes for urate oxidation in 341 prokaryotic species (see Fig. 1c) classified based on the presence of a single (monoderm) or a double (diderm) cell membrane^40^.

We have described the identification of a protein family of unknown function (COG3478) as an integral membrane, cytochrome *c* urate oxidase (PuuD). This identification was initially suggested by the analysis of the gene association network (Fig. 1b) and then confirmed by the analysis of gene distribution (Fig. 1c), as quantitatively evaluated through the use of association rules (Table 1). This method, inspired by the logic extension^28^ of the widely used correlated and anti-correlated phylogenetic profiles^26^,^27^, can be useful in the presence of functional associations not adequately described by pairwise relations, as exemplified here. The same analysis that enabled the functional assignment of COG3748, also provides evidence that the identification of PuuD completes the genetic repertoire of enzymes involved in urate oxidation. There is a very small number of cases in which any of the four urate oxidation genes is found in the absence of *urah* and *urad*, suggesting that there are no alternative genes encoding these enzymatic activities; on the other hand, among the 1689 different species considered there are only twelve cases in which the presence of *urah* and *urad* is not explained by the presence of *uox*, *hpxO*, *hpyO*, or *puuD* genes (Table 1). Most of these exceptions can be explained by errors of the gene identification procedure (Supplementary Table S1). Genes encoding PuuD urate oxidase have a peculiar organism distribution, being found only in aerobic bacteria with two cell membranes -diderms, approximately corresponding to gram-negative in the traditional classification^40^. In these organisms, PuuD is the prevalent form of urate oxidase, while Uox is prevalent in monoderm (~gram-positive) prokaryotes (Fig. 5).

A genetic approach was selected for the experimental validation of the activity of PuuD proteins predicted by bioinformatics. The expression and purification of integral membrane proteins is notoriously challenging, and we did not observe protein overexpression when the gene was cloned in a heterologous (*E. coli*) expression system (Supplementary Fig. S11). Furthermore, the presence of a cytochrome *c* domain suggested that the enzymatic activity of this protein could depend on other components of an electron transfer chain. Ultimately, the results obtained with deleted and complemented strains along with bioinformatics provide conclusive evidence for the assignment of a urate oxidase activity to COG3478.

PuuD proteins are characterized by the presence of DUF989, an integral membrane domain with 8 TM helices. Although this organization can be reminiscent of transporter or channel proteins, the evidence presented here indicates that the domain is involved in urate oxidation and not in urate transport. Elements of evidence are the frequent genomic association of PuuD protein with known urate transporters (see e.g. Supplementary Fig. S1) and the complete rescue of the phenotype obtained through complementation of the PuuD deletion mutant with a cytosolic Uox. The analysis of gene context and gene distribution (see Fig. 1c) indicates that all full-length DUF989 proteins have a role in purine degradation; these proteins additionally contain a cytochrome *c* domain. Included in the list of DUF989 members in Pfam is a group of shorter proteins characterized by a partial match to the domain HMM and the absence of the C-terminal cytochrome. These shorter DUF989 proteins contain 4 TM helices corresponding to helices I, II, V, and VI of PuuD (Supplementary Fig. S12). This suggests that helices I, II, V, and VI - the most conserved elements in the PuuD alignment (see Fig. 3) - constitute the core of the DUF989 fold. Genes encoding the shorter DUF989 variant have a genetic context completely different from that of *puuD* genes (Supplementary Fig. S13). Searches based on HMM-HMM comparisons do not reveal any similarity of DUF989 with known protein domains. A distant similarity is found (E-vaule = 0.15; P-value = 10^−6^) with DUF2269, an integral membrane domain of unknown function.

PuuD proteins are also characterized by the presence of the heme-binding cytochrome *c* domain. Cytochrome *c* is an extremely widespread electron transfer domain that mostly occurs in single-domain proteins. However, as in the case of PuuD, it is also often found fused with other protein domains. The association with DUF989 is the third most common multi-domain architecture involving cytochrome *c* and another Pfam domain. The most common association is with the cytochrome CBB3 domain as is found in ubiquinol-cytochrome *c* reductases^41^. Other common associations are with the heme binding CCP_MauG domain as is found in di-heme cytochrome *c* peroxidases^42^, and with the COX2 domain as is found in cytochrome *c* oxidases^43^. All the cited examples are integral membrane or periplasmic oxidoreductases in which the electron transfer activity of cytochrome *c* is an essential component of the enzyme catalysis. The presence of a cytochrome *c* domain in PuuD urate oxidases explains the otherwise puzzling observation that in *Sinorhizobium meliloti*—a PuuD-encoding organism (see Fig. 1c)—mutations of the *ccmC* gene coding for an integral membrane heme exporter^44^ impair the ability to use purines as nitrogen source^45^. As a protein with a signal anchor, PuuD will be directed to the membrane in an unfolded state, implying that the heme must be assembled with the apo-Cyt_c domain in the extra-cytoplasmic space. Together with the notion that cytochrome *c* proteins typically function outside the plasma membrane in prokaryotes, the purine degradation deficient phenotype of *ccmC* mutants provides additional support for the N_out_-C_out_ topology of PuuD proteins (see Fig. 3b). According to this topology, conserved extra-membrane regions are exposed towards the cytosol, suggesting that urate oxidation by PuuD takes place on the cytosolic side of the membrane. The electrons removed from the urate molecule could be transferred to the extra-cellular (periplasmic) Cyt_c domain through physical interaction with the transmembrane DUF989 domain. The hypothesis of an oxidation reaction catalysed by DUF908 on the cytosolic side is also consistent with the cytosolic localization of the enzymes (Urah and Urad) acting on the metastable products of urate oxidation.

The identification of an integral membrane urate oxidase employing a *c*-type heme suggests an unsuspected link between purine catabolism and bacterial respiration. However, we did not observe specific inhibition of the PuuD activity in the presence of a classical inhibitor of the electron transport chain. Azide was not expected to inhibit urate utilization through direct binding to the PuuD protein, but through binding to the cytochrome *c* oxidase, the terminal acceptor of the respiratory electron chain. The interpretation of the results obtained with the azide inhibitor is complicated by the fact that bacteria have branched respiratory pathway with multiple terminal oxidases exhibiting different sensitivity to the typical respiratory inhibitors^46^,^47^. Another complication arises from the known ability of azide to bind the heme cofactor of catalase^48^, which is expected also to affect the activity of the H_2_O_2_-producing zfUox. An alternative hypothesis to the electron transfer can be put forward by considering the known ability of cytochrome *c* to act as a scavenger of the superoxide ion (O_2_^−^)^49^. The oxidation of the oxopurines hypoxanthine and xanthine, by xanthine oxidase (XO), can produce superoxide according to the reaction^50^: RH + H_2_O + 2O_2_ ⇋ ROH + 2O_2_^−^ + 2H^+^. If the PuuD reaction mechanism imitates that of the preceding enzyme in the pathway, the presence of the cytochrome *c* domain would be justified by the scavenging of a reactive oxygen species produce by urate oxidation. In this hypothesis, however, the cytochrome *c* domain would have an accessory function, while the evidence of the invariant presence of this domain in PuuD proteins and the phenotype of the *ccmC* mutant rather suggest that the cytochrome is an essential component of the enzyme.

With four protein families and three different reaction mechanisms, urate oxidase provides an illustrative example of the variety of solutions that can be found in nature for the same biochemical problem^51^. Among these independent inventions, the cofactor-less Uox, which is found in all three domains of life (see Fig. 1c), is probably the most ancient solution, but not necessarily the best one. It appears an ideal solution for an enzyme localized in a single-membrane compartment specialized in the detoxification of reactive oxygen species. PuuD, with its more restricted organism distribution, was apparently invented later in a diderm ancestor (possibly a proteobacteria) through a gene fusion of a preexisting integral membrane domain with a cytochrome *c* (see Fig. 3 and Supplementary Fig. S9). This invention likely provided a selective advantage to diderm bacteria, as suggested by the observation that in these organisms PuuD has almost completely replaced Uox as urate oxidase.

## Methods

### Analysis of gene associations

The predicted association network for genes involved in urate oxidation was obtained by interrogating the String database (http://string-db.org) with a list of Cluster of Orthologous Groups (COG3648, COG2351, and COG3195) corresponding, respectively, to the *uox*, *urah*, and *urad* gene families. The “gene neighborhood”, “gene fusion” and “gene co-occurrence” evidence were used to predict functional associations. To analyse the distribution of genes involved in urate oxidation in complete proteomes, homology searches where performed using Hidden Markov Models of the corresponding protein families in Pfam (see Supplementary Table S1) and the Hmmsearch program^52^. The results for each family were clustered using Blast best reciprocal hits (BRH)^23^ and the MCL program^53^ with an inflation index of 3. Genes clusters comprising reference genes involved in purine degradation (see Supplementary Table S1) were considered. The entire procedure was automated using Perl scripts. In the case of *hpxO* and *hpyO*, genes belonging to large families containing many paralogs, the clusters were further refined through phylogenetic analysis (Supplementary Fig. S14). The representation of gene distributions along the species phylogeny (see Fig. 1c) was obtained with the R software using the Ape library^54^ and the heatmap.phylo function (Johan Renaudie; http://stackoverflow.com/questions/15153202).

Association rules^20^ based on gene distributions were determined using the Apriori program^21^ using as input vectors of a gene distribution and its logical negation. Rules implicating COG3748 as consequent (see Table 1) were ranked according to their confidence^20^, defined as the number of cases in which the rule is correct relative to the number of cases in which it is applicable. The significance of the dependency of the antecedent and consequent of a rule was computed through the Fisher’s Exact Test. Note that significance is not an indication of the correctness of the logic implication, as the opposite of a rule will receive the same p-value.

### Sequence and structure analysis

The ClustalW program was used for multiple sequence alignments and tree reconstruction (based on neighbor-joining clustering of Kimura-corrected distances). Domain analysis was conducted using Pfam^36^ and HHpred^37^. Figures of multiple alignments decorated with structural elements were obtained with the Espript web server^55^. Transmembrane topology was determined with Phobius^56^ and TopCons^57^. The representation of membrane topology of PuuD proteins were obtained with the Protter web server^58^. Homology modelling was performed using Swiss Model (http://swissmodel.expasy.org/) and the Pymol program was used to analyse structural models.

### Deleted and complemented strains

The deletion of *Atu2314* gene of *A. fabrum* C58 was obtained with a described procedure^30^. Briefly, the upstream (1800 bp.) and the downstream (1748 bp.) sequences of Atu2314 gene were cloned into pJQ200sk plasmid (ATCC: 77483) previously digested with SacI and BamHI restriction enzymes. The pJQΔUox2 plasmid was confirmed by sequencing and used to transform *A. fabrum* C58 cells by electroporation. The recombinant cells were selected by growth on gentamicin-supplemented LB agar plate and then on sucrose-supplemented LB agar plate. Deletion of Atu2314 was confirmed by sequencing. The zfUox complemented strain was obtained by transforming ΔAtu2314 with a pSRKGm plasmid^31^ modified by the insertion of the coding sequence of the *uox* gene from zebrafish (ZDB-GENE-030826-24) within NdeI and BamHI restriction sites. The zfUox CDS was subcloned from a previously generated pET11b expression vector (Ramazzina, I., unpublished). The presence of the zfUox CDS into the pSRKGm plasmid was confirmed by sequencing.

### Bacterial growth

*A. fabrum* C58 strains were first growth on LB medium at 28 °C. After 20 h, bacteria were spun down, washed with M9 minimal medium without any nitrogen and carbon sources and diluted to an optical density at 600 nm (OD_600_) of 0.05 in M9 minimal medium with NH_4_Cl (0.02%) and arabinose (0.2%) as sole nitrogen and carbon sources. The cultures were grown for another 20 h, spun down, washed twice with M9 minimal medium without any nitrogen or carbon sources and then diluted to an OD_600_ of about 0.1 in M9 minimal medium containing arabinose (0.2%) plus NH_4_Cl (0.02%) or uric acid (0.02%), with the addition of IPTG 1 mM where no differently specified. Cultures were incubated with shaking at 28 °C. At intervals, OD_600_ measurements were taken (Ultraspec 2000, Pharmacia Biotech).

### Urate utilization assays

Bacteria inoculated at OD_600_ 0.05 were grown in M9 medium supplemented with arabinose and NH_4_Cl as described. After 20 h, cells were spun down, washed twice with M9 medium without nitrogen or carbon sources and resuspended to an OD_600_ ~1 in M9 medium containing arabinose and without any nitrogen sources. After 90 minutes, urate (0.02%) was added to the cultures, together with IPTG 1 mM where no differently specified. At intervals, 1 ml of culture was spun down and 0.1 ml of supernatant was diluted with 0.9 ml H_2_O to measure the absorbance at 293 nm (Cary 50, Varian). Urate utilization was measured in the absence and in the presence of different concentrations of sodium azide.

To measure soluble urate oxidase activity, an aliquot of each culture was taken after 5 hours after the addition of urate and IPTG, and the cells were resuspended in 50 mM NaP pH 7.0 containing 1 mM PMSF and lysed by sonication. After centrifugation (7500 rpm for 15 min), the protein concentration of the soluble fraction was determined using the Bradford colorimetric assay, with bovine serum albumin as standard. Cell-free extracts (250 μg) were added to 0.1 ml solutions containing 0.11 mM urate and the reaction was monitored by following the decrease in absorbance at 293 nm.

### Catalase activity and oxidative stress assay

Cells were grown starting from OD_600_ ~0.1 in M9 medium containing arabinose (0.02%) and NH_4_Cl (0.02%) or urate (0.02%) with the addition of IPTG 1 mM where no differently specified. After 12 hours, cells were spun down and the cell-free extracts were obtained as described above. The catalase activity of each sample was measured spectrophotometrically by following the decrease of absorbance at 240 nm^59^. One unit of enzyme was defined as the amount of enzyme catalysing the turnover of 1 μmol of substrate per minute under the assay conditions. To measure the resistance of different strains to H_2_O_2_, cells grown in urate for 12 h were spun down, washed once with 50 mM sodium phosphate buffer pH 7.0 and diluted in the same buffer to an OD_600_ of 0.1. Progressive dilutions (1:5) of the cells were spotted on LB agar plates with different H_2_O_2_ concentration and incubated overnight at 28 °C.

### PuuD cloning and expression

A PuuD expression vector was constructed by cloning the Atu2314 (PuuD) coding sequence into the pET11b vector. The Atu2314 coding sequence was amplified from *A. fabrum* strain C58 genomic DNA using Taq DNA polymerase with primer forward 5′-CATATGTACGAATACGCCATTGC and primer reverse 5′-GGATCCCACGCAGCAGAACC in standard reaction conditions. The PCR product was cloned in pGEM-T easy vector (Promega), which was then digested with NdeI and BamHI (Thermo Scientific) and ligated to a linearized pET11b vector DNA with compatible ends. The correct insertion of the PuuD coding sequence was verified by sequencing (Supplementary Fig. S11). Expression attempts were conducted in transformed BL21 cells (Novagene). Different induction conditions with IPTG 1 mM were tested, from 4 to 16 hours and from 20 °C to 37 °C.

## Additional Information

**How to cite this article**: Doniselli, N. *et al.* The identification of an integral membrane, cytochrome *c* urate oxidase completes the catalytic repertoire of a therapeutic enzyme. *Sci. Rep.* **5**, 13798; doi: 10.1038/srep13798 (2015).

## Supplementary Material

Supplementary Information

Supplementary Dataset

## Figures and Tables

**Table 1:** Association rules implicating COG3748 as consequent.

**Association rule^a^**	**TT**	**TF**	**FT**	**FF**	**Confidence**	**P**
Urah Urad !Uox !HpxO !HpyO - > COG3748	154	12	5	1519	0.928	1 × 10^−196^
Urad !Uox !HpxO !HpyO - > COG3748	155	26	4	1505	0.856	1 × 10^−185^
Urad !Uox !HpxO - > COG3748	155	44	4	1487	0.779	2 × 10^−172^
Urad !Uox - > COG3748	157	95	2	1436	0.623	6 × 10^−151^
Urad - > COG3748	158	272	1	1259	0.367	1 × 10^−103^
!Uox - > COG3748	158	1339	1	192	0.106	1 × 10^−8^

^a^Best pairwise and higher-order association rules involving genes for urate oxidation. Genes are connected by logical ‘and’ operators; exclamation marks indicate the logical ‘not’ operator. The truth table reports the number of cases in which the antecedent and the consequent of a rule are true (T) or false (F).
