# Supplementary material for: The identification of an integral membrane, cytochrome c urate oxidase completes the catalytic repertoire of a therapeutic enzyme
Source: Sci Rep. 2015 Sep 8;5:13798. doi: 10.1038/srep13798 (PMC4562309; doi:10.1038/srep13798)
Supplement: Supplementary Information [file srep13798-s1.pdf]

The identification of an integral membrane, cytochrome *c* urate oxidase completes the catalytic repertoire of a therapeutic enzyme. Nicola Doniselli, Enrico Monzeglio, Alessandro Dal Palù, Angelo Merli, Riccardo Percudani.

Supplementary figures

Supplementary Figure S1. **Predicted COG associations to genes involved in urate oxidation.**

Supplementary Figure S2. **Neighborhood of COG3748 genes in selected genomes**.

Supplementary Figure S3. **Map of urate oxidation incapacity in complete genomes**.

Supplementary Figure S4. **Uric acid utilization by pre-induced cultures**.

Supplementary Figure S5. **Enzymatic activity of recombinant zfUox**.

Supplementary Figure S6. **Phylogeny of COG3748 proteins**.

Supplementary Figure S7. **Multiple alignment of representative PuuD proteins**.

Supplementary Figure S8. **Position and topology of transmembrane helices in PuuD proteins**.

Supplementary Figure S9. **3D homology model of the PuuD Cyt_c domain.**

Supplementary Figure S10. **Urate utilization in the presence of sodium azide**.

Supplementary Figure S11. **PuuD cloning and overexpression attempts**.

Supplementary Figure S12. **Comparison of PuuD with the short DUF989 variant**.

Supplementary Figure S13. **Neighborhood of genes encoding the short DUF989 variant**.

Supplementary Figure S14. **Selection of HpxO and HpyO proteins in the family phylogeny**.

Your Input:

(Urah)	COG2351	Transthyretin-like protein	
(Urad)	COG3195	Uncharacterized protein conserved in bacteria	
(Uox)	COG3648	Uricase (urate oxidase)	

Predicted Functional Partners:

			Neighborhood	Gene Fusion	Cooccurrence	Coexpression	Experiments	Databases	Textmining	Score
	COG2233	Xanthine/uracil permeases	•	•	•	x	x	x	x	0.816
	COG0726	Predicted xylanase/chitin deacetylase	•	•		x	x	x	x	0.802
	COG0624	Acetylornithine deacetylase/Succinyl-diaminopimelate desuccinylase and related deacylases	•	•		x	x	x	x	0.685
Candidate urate oxidase →	NOG75554	non supervised orthologous group	•	•		x	x	x	x	0.669
	COG3748	Predicted membrane protein	•		•	x	x	x	x	0.658
	COG4631	Xanthine dehydrogenase, molybdopterin-binding subunit B	•		•	x	x	x	x	0.611
	COG4630	Xanthine dehydrogenase, iron-sulfur cluster and FAD-binding subunit A	•		•	x	x	x	x	0.593
HpyO →	COG2072	Predicted flavoprotein involved in K+ transport	•	•	•	x	x	x	x	0.592
	COG3194	Ureidoglycolate hydrolase	•		•	x	x	x	x	0.572
	COG4266	Allantoicase	•		•	x	x	x	x	0.527
	COG0402	Cytosine deaminase and related metal-dependent hydrolases	•		•	x	x	x	x	0.524
	COG1975	Xanthine and CO dehydrogenases maturation factor, XdhC/CoxF family	•			x	x	x	x	0.490
	NOG249698	non supervised orthologous group	•			x	x	x	x	0.431
	COG3257	Uncharacterized protein, possibly involved in glyoxylate utilization	•		•	x	x	x	x	0.414
	COG3960	Glyoxylate carboligase	•		•	x	x	x	x	0.389
	COG1953	Cytosine/uracil/thiamine/allantoin permeases	•		•	x	x	x	x	0.381
	COG0583	Transcriptional regulator	•			x	x	x	x	0.369
	COG1802	Transcriptional regulators	•			x	x	x	x	0.360
	COG0044	Dihydroorotase and related cyclic amidohydrolases	•			x	x	x	x	0.353
	COG3622	Hydroxypyruvate isomerase	•		•	x	x	x	x	0.341
	NOG145560	non supervised orthologous group	•			x	x	x	x	0.338
	COG4126	Hydantoin racemase	•		•	x	x	x	x	0.327
	COG2080	Aerobic-type carbon monoxide dehydrogenase, small subunit CoxS/CutS homologs	•			x	x	x	x	0.326
	NOG40242	non supervised orthologous group	•			x	x	x	x	0.316
	COG0075	Serine-pyruvate aminotransferase/archaeal aspartate aminotransferase	•			x	x	x	x	0.297
	COG1529	Aerobic-type carbon monoxide dehydrogenase, large subunit CoxL/CutL homologs	•			x	x	x	x	0.296
	COG1319	Aerobic-type carbon monoxide dehydrogenase, middle subunit CoxM/CutM homologs	•			x	x	x	x	0.291
	COG2508	Regulator of polyketide synthase expression	•			x	x	x	x	0.291
	COG0642	Signal transduction histidine kinase	•			x	x	x	x	0.286
	NOG44195	non supervised orthologous group	•			x	x	x	x	0.282
	COG0745	Response regulators consisting of a CheY-like receiver domain and a winged-helix DNA-binding do [...]	•			x	x	x	x	0.277
	COG2379	Putative glycerate kinase	•			x	x	x	x	0.276
HpxO →	COG0654	2-polyprenyl-6-methoxyphenol hydroxylase and related FAD-dependent oxidoreductases	•		•	x	x	x	x	0.273
	NOG44735	non supervised orthologous group	•			x	x	x	x	0.271
	COG0590	Cytosine/adenosine deaminases	•			x	x	x	x	0.268
	COG2084	3-hydroxyisobutyrate dehydrogenase and related beta-hydroxyacid dehydrogenases	•		•	x	x	x	x	0.265
	NOG06493	non supervised orthologous group	•			x	x	x	x	0.263
	NOG124263	non supervised orthologous group	•			x	x	x	x	0.254
	NOG112896	non supervised orthologous group	•			x	x	x	x	0.247
	COG1309	Transcriptional regulator	•			x	x	x	x	0.238

Supplementary Figure S1. **Predicted COG associations to genes involved in urate oxidation.** Output of the String database obtained using COG3648 (Uox), COG2351 (Urah), and COG3195 (Urad) as a query and selecting “gene neighborhood”, “gene fusion” and “gene co-occurrence” as association evidence.

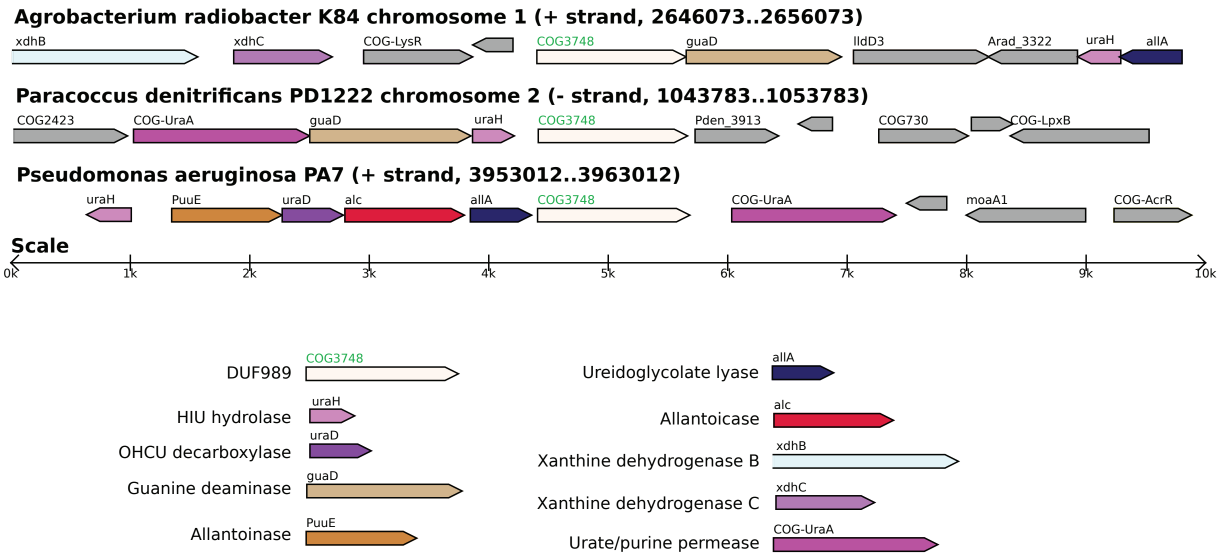

Supplementary Figure S2. **Neighborhood of COG3748 genes in selected genomes**. A region of 10 Kb around the gene encoding COG3748 is shown for *A. radiobacter*, *P. denitrificans*, *P. aeruginosa*. Genes involved in purine degradation are shown in color as indicated by the figure legend. Note the absence of known genes encoding urate oxidase in the genomic clusters. Evidence of genetic linkage between allantoinase (puuE) and urate oxidase (puuD) was previously reported in *P. aeruginosa* [34]. Gene neighborhood was obtained with the Microbesonline web server (http://www.microbesonline.org).

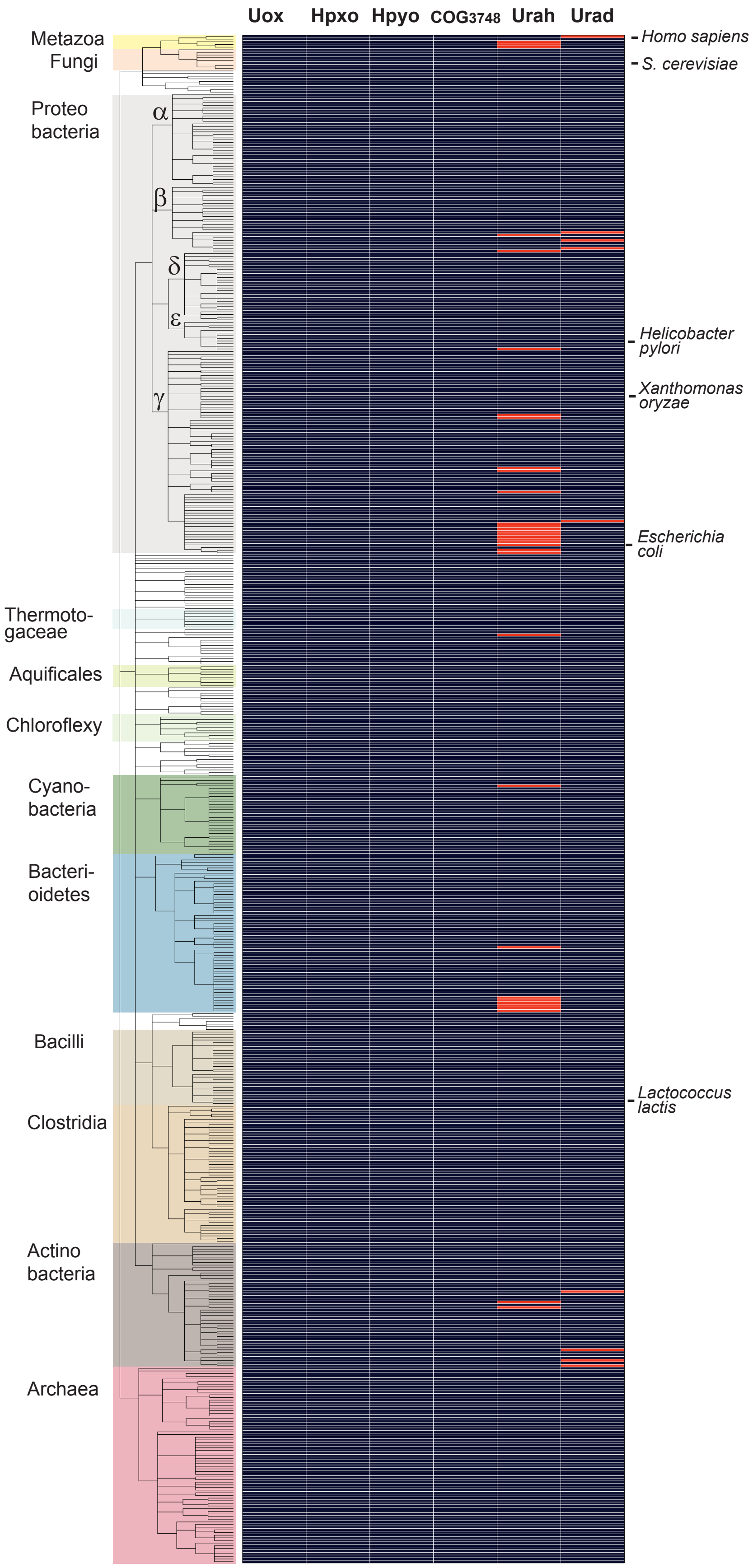

Supplementary Figure S3. **Map of urate oxidation incapacity in complete genomes**. The tree represents 571 distinct genera on a total of 1264 distinct species not possessing either the *uox*, *hpxo*, *hpyo*, or COG3748 genes and/or both the *urah* and *urad* genes. The presence (red) or the absence (blue) of genes is shown alongside the organism tree. Main taxonomic groups and organisms discussed in the text are indicated.

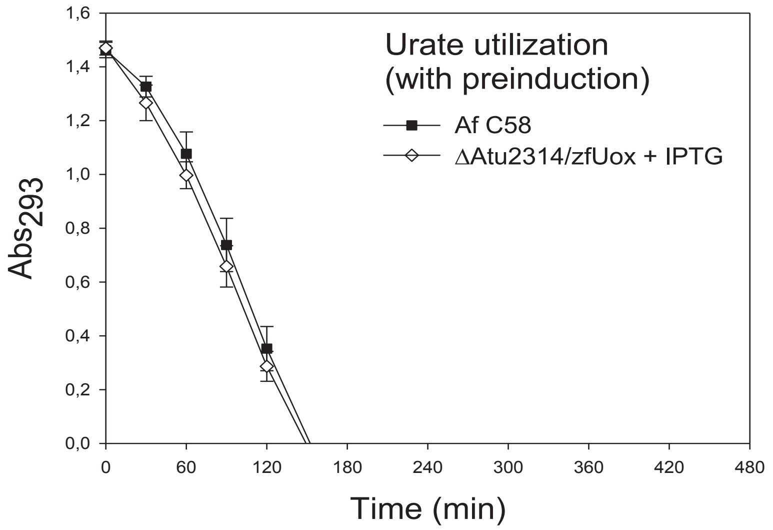

Supplementary Figure S4. **Uric acid utilization by pre-induced cultures**. Cells of *A. fabrum* C58 and ΔAtu2314/zfUox grown for 10 h with urate as nitrogen source were resuspended in fresh medium containing 0.02% urate. Error bars represent standard deviations obtained from three independent experiments.

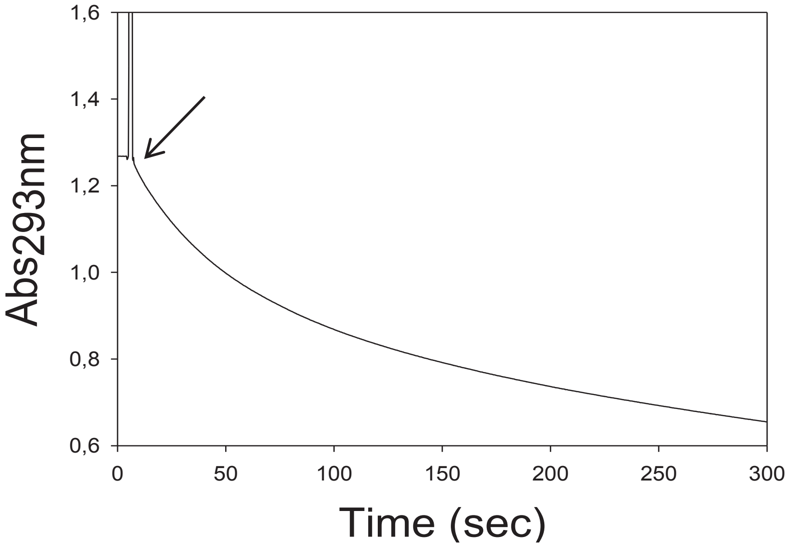

Supplementary Figure S5. **Enzymatic activity of recombinant zfUox**. Degradation of urate (0.11mM) in NaPi buffer, pH 7.6, catalysed by 5 µg of purified zfUox (pET11b expression system) as monitored by the decrease in absorbance at 293 nm. The arrow indicates the addition of the enzyme to the urate solution

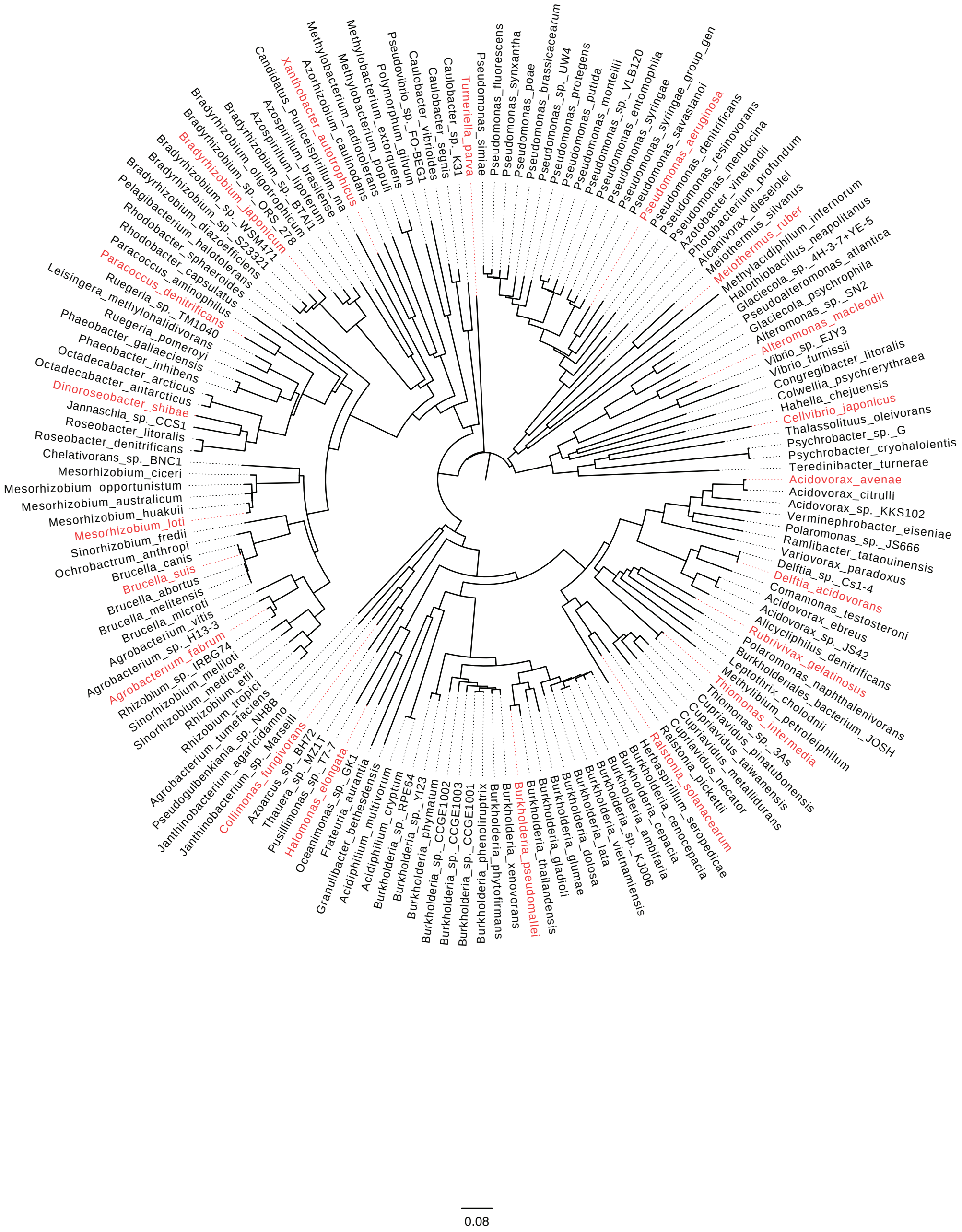

Supplementary Figure S6. **Phylogeny of COG3748 proteins**. The midpoint-rooted phylogenetic tree has been obtained with neighbor-joining using Kimura-corrected genetic distances. Sequences selected for the analysis of residue conservation are shown in red. Database accession numbers are reported in Table S1.

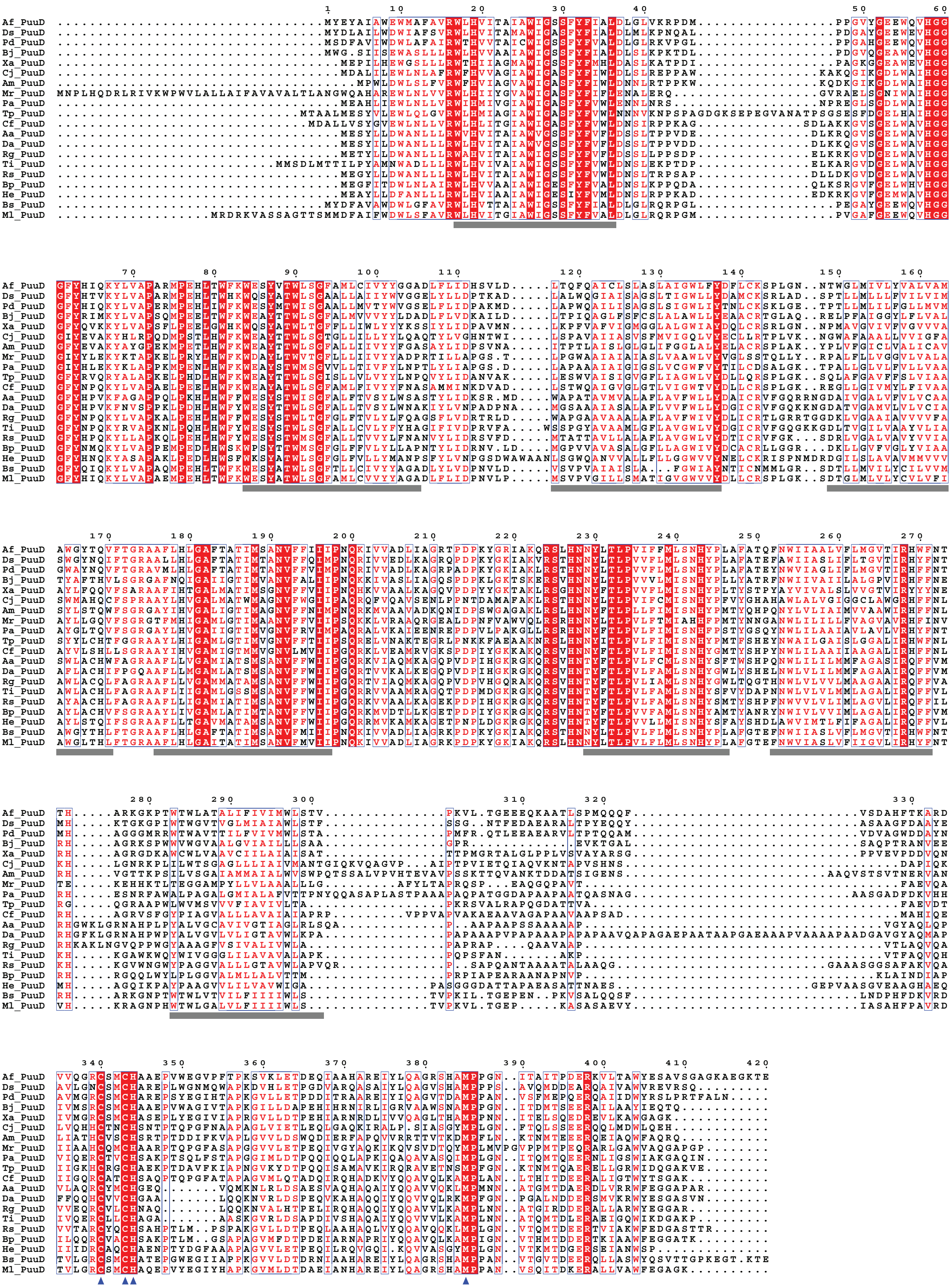

Supplementary Figure S7. **Multiple alignment of representative PuuD proteins**. Sequences are indicated with abbreviated species names; full names and database accession numbers are reported in Fig. S4 and Table S1. The identified transmembrane helices in AfPuuD are indicated by gray bars. Blue arrowheads indicate residues supposedly involved in heme coordination.

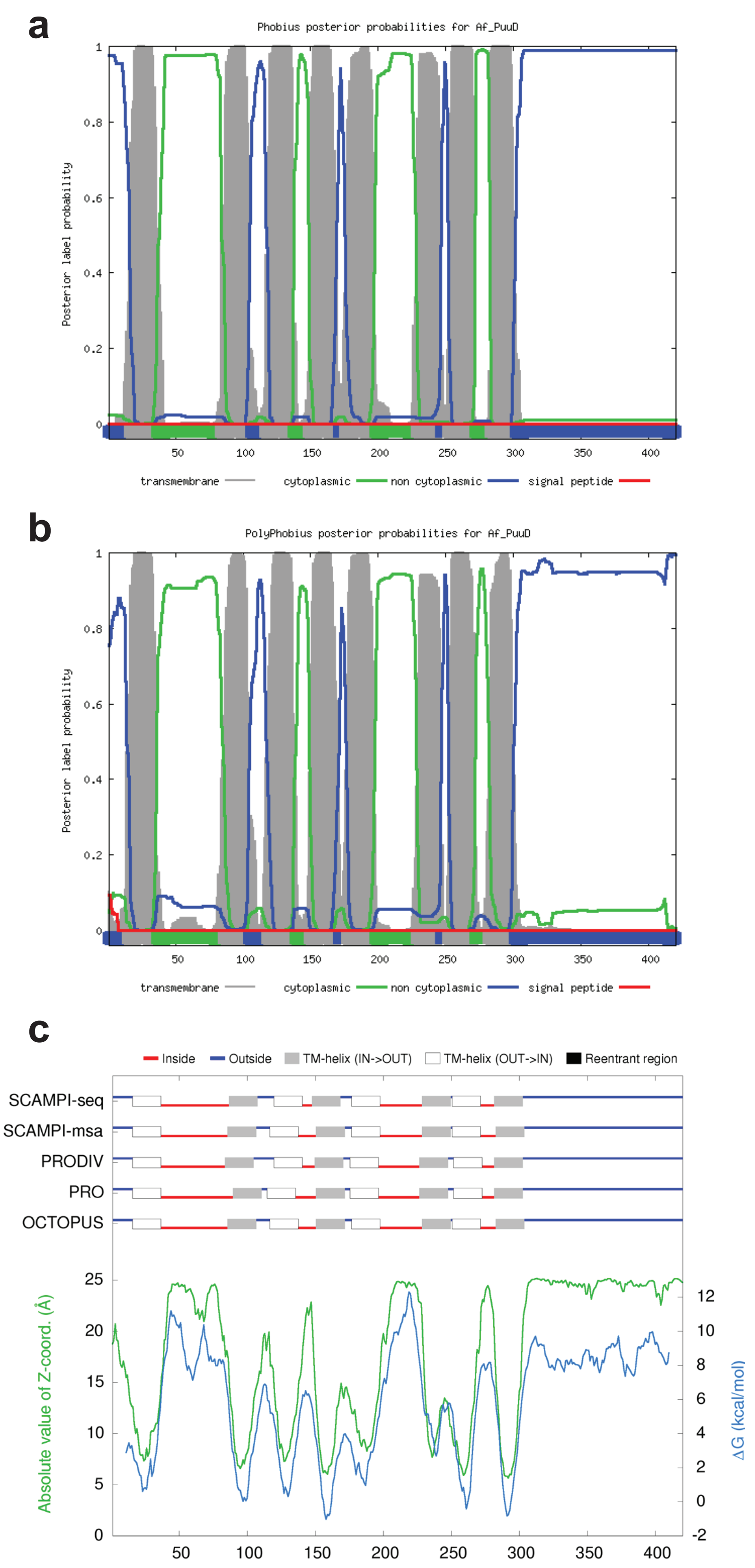

Supplementary Figure S8. **Position and topology of transmembrane helices in PuuD proteins**. (a) Phobius output obtained with the AfPuuD protein. (b) Phobius output obtained with the multiple alignment reported in Fig. S5. (c) TopCons analysis of the AfPuuD protein showing the consensus of different predictors.

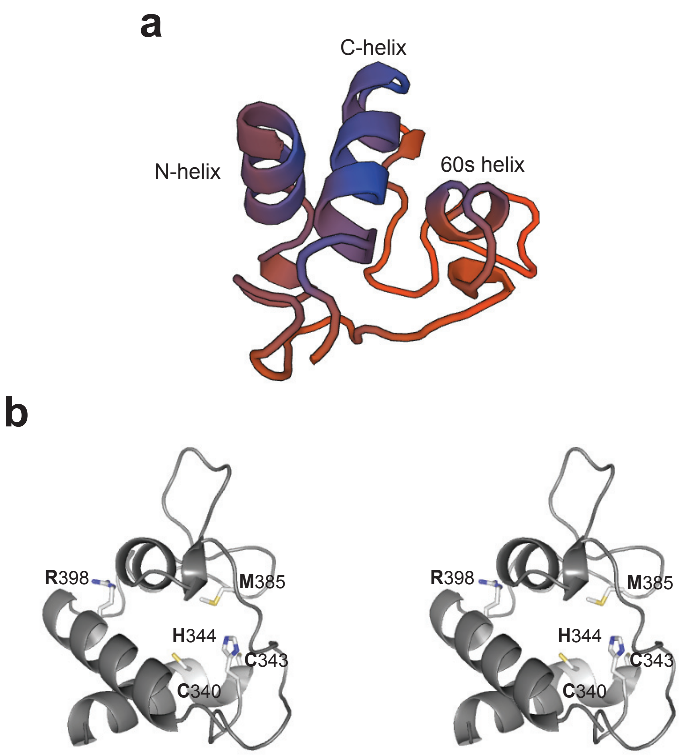

Supplementary Figure S9. **3D homology model of the PuuD Cyt_c domain.** The structural model (GMQE=0.47; QMEAN4=-4.09) is based on the comparison of the Cyt c region of AfPuuD (aa 329-410) with the template PDB structure 2d0w. (a) Cartoon representation of the structure colored according to the model confidence from blue (high) to red (low); helices are named according to the mitochondrial cytochrome c notation. (b) Stereoview showing the position of the heme binding residues and the invariant Arg in the sequence alignment.

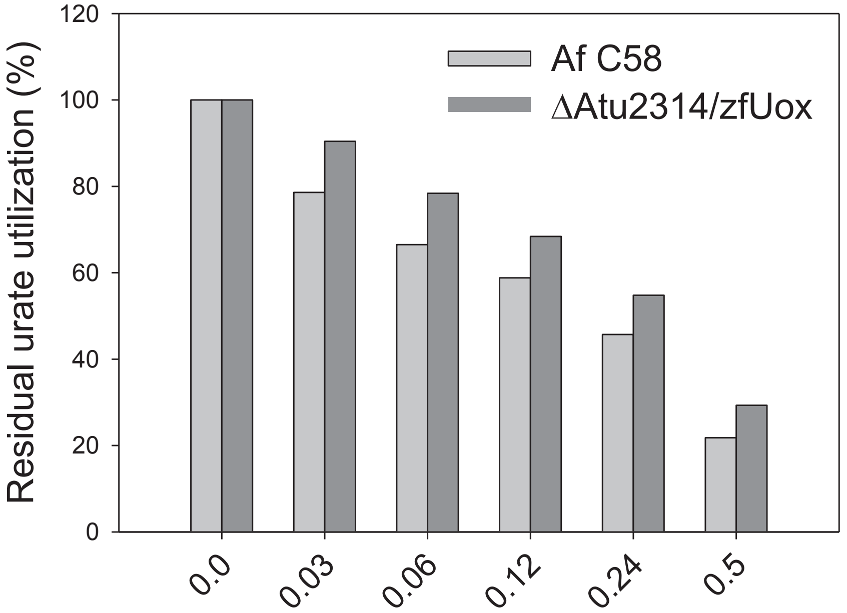

Supplementary Figure S10. **Urate utilization in the presence of sodium azide**. Comparison of urate utilization in the absence or in the presence of increasing concentrations of sodium azide. Activity was measured as decrease of absorbance at 293 nm at 2h after the addition of urate to intact cells.

a

```
                                   rbs        NdeI
pET11-PuuD AATAATTTTGTTTAACTTTAAGAAGGAGATATACATATGTACGAATACGCCATTGCGTGGGAATGGATGGCCTTTGCCGTCCGCTGGCTCCACGTCATCACCGCCATCGC
Atu2314    -----------------------------------ATGTACGAATACGCCATTGCGTGGGAATGGATGGCCTTTGCCGTCCGCTGGCTCCACGTCATCACCGCCATCGC
                                               M  Y  E  Y  A  I  A  W  E  W  M  A  F  A  V  R  W  L  H  V  I  T  A  I  A

CTGGATCGGCTCATCCTTTTATTTCATCGCGCTCGATCTCGGGCTGGTCAAACGCCCGGATATGCCGCCAGGCGTCTATGGCGAGGAGTGGCAGGTGCATGGCGGCGGCTTTTACCACAT
CTGGATCGGCTCATCCTTTTATTTCATCGCGCTCGATCTCGGGCTGGTCAAACGCCCGGATATGCCGCCAGGCGTCTATGGCGAGGAGTGGCAGGTGCATGGCGGCGGCTTTTACCACAT
 W  I  G  S  S  F  Y  F  I  A  L  D  L  G  L  V  K  R  P  D  M  P  P  G  V  Y  G  E  E  W  Q  V  H  G  G  G  F  Y  H  I

CCAGAAATATCTGGTCGCTCCAGCCCGCATGCCCGAGCACCTGACATGGTTCAAATGGGAAAGCTATGTCACCTGGCTTTCCGGCTTCGCCATGCTCTGCATCGTTTATTACGGCGGCGC
CCAGAAATATCTGGTCGCTCCAGCCCGCATGCCCGAGCACCTGACATGGTTCAAATGGGAAAGCTATGTCACCTGGCTTTCCGGCTTCGCCATGCTCTGCATCGTTTATTACGGCGGCGC
 Q  K  Y  L  V  A  P  A  R  M  P  E  H  L  T  W  F  K  W  E  S  Y  V  T  W  L  S  G  F  A  M  L  C  I  V  Y  Y  G  G  A

GGACCTTTTCCTCATCGACCATTCGGTGCTTGATCTCACCCAGTTCCAGGCCATCTGTCTGTCATTGGCCTCGCTCGCCATCGGCTGGCTGTTTTACGATTTCCTTTGCAAATCACCGCT
GGACCTTTTCCTCATCGACCATTCGGTGCTTGATCTCACCCAGTTCCAGGCCATCTGTCTGTCATTGGCCTCGCTCGCCATCGGCTGGCTGTTTTACGATTTCCTTTGCAAATCACCGCT
 D  L  F  L  I  D  H  S  V  L  D  L  T  Q  F  Q  A  I  C  L  S  L  A  S  L  A  I  G  W  L  F  Y  D  F  L  C  K  S  P  L

CGGCAACAATACCTGGGGCCTGATGATCGTGCTCTATGTCGCGCTGGTGGCGATGGCATGGGGTTATACGCAGGTTTTCACCGGCCGCGCCGCCTTCCTGCATCTCGGCGCCTTCACCGC
CGGCAACAATACCTGGGGCCTGATGATCGTGCTCTATGTCGCGCTGGTGGCGATGGCATGGGGTTATACGCAGGTTTTCACCGGCCGCGCCGCCTTCCTGCATCTCGGCGCCTTCACCGC
 G  N  N  T  W  G  L  M  I  V  L  Y  V  A  L  V  A  M  A  W  G  Y  T  Q  V  F  T  G  R  A  A  F  L  H  L  G  A  F  T  A

CACCATCATGTCGGCGAACGTATTCTTCATCATCATCCCCAACCAGAAGATCGTCGTGGCCGACCTGATCGCCGGGCGCACGCCCGACCCGAAATATGGCCGCATCGCCAAGCAGCGCTC
CACCATCATGTCGGCGAACGTATTCTTCATCATCATCCCCAACCAGAAGATCGTCGTGGCCGACCTGATCGCCGGGCGCACGCCCGACCCGAAATATGGCCGCATCGCCAAGCAGCGCTC
 T  I  M  S  A  N  V  F  F  I  I  I  P  N  Q  K  I  V  V  A  D  L  I  A  G  R  T  P  D  P  K  Y  G  R  I  A  K  Q  R  S

ATTGCACAACAACTACCTGACGCTGCCCGTCATCTTCTTCATGCTGTCGAACCATTACCCGCTGGCCTTTGCGACGCAATTCAACTGGATCATCGCAGCGCTTGTCTTCCTGATGGGTGT
ATTGCACAACAACTACCTGACGCTGCCCGTCATCTTCTTCATGCTGTCGAACCATTACCCGCTGGCCTTTGCGACGCAATTCAACTGGATCATCGCAGCGCTTGTCTTCCTGATGGGCGT
 L  H  N  N  Y  L  T  L  P  V  I  F  F  M  L  S  N  H  Y  P  L  A  F  A  T  Q  F  N  W  I  I  A  A  L  V  F  L  M  G  V

CACCATCCGCCACTGGTTCAACACAACCCATGCCCGCAAGGGCAAGCCGACATGGACCTGGCTTGCGACCGCCCTCATCTTCATCGTCATCATGTGGCTTTCCACCGTGCCGAAGGTGCT
CACCATCCGCCACTGGTTCAACACAACCCATGCCCGCAAGGGCAAGCCGACATGGACCTGGCTTGCGACCGCCCTCATCTTCATCGTCATCATGTGGCTTTCCACCGTGCCGAAGGTGCT
 T  I  R  H  W  F  N  T  T  H  A  R  K  G  K  P  T  W  T  W  L  A  T  A  L  I  F  I  V  I  M  W  L  S  T  V  P  K  V  L

GACCGGGGAAGAGGAACAGAAGGCCGCCACTCTCTCCCCCATGCAGCAGCAATTCGTCAGCGACGCCCATTTCACCAAGGCCCGCGATGTGGTTCAGGGCCGCTGTTCCATGTGCCACGC
GACCGGGGAAGAGGAACAGAAGGCCGCCACTCTCTCCCCCATGCAGCAGCAATTCGTCAGCGACGCCCATTTCACCAAGGCCCGCGATGTGGTTCAGGGCCGCTGTTCCATGTGCCACGC
 T  G  E  E  E  Q  K  A  A  T  L  S  P  M  Q  Q  Q  F  V  S  D  A  H  F  T  K  A  R  D  V  V  Q  G  R  C  S  M  C  H  A

GGCCGAGCCGGTGTGGGAAGGCGTGCCCTTCACGCCAAAATCCGTGAAGCTGGAAACCGATGAACAGATCGCCGCCCATGCGCGCGAAATCTATTTGCAAGCCGGCCGCAGCCATGCCAT
GGCCGAGCCGGTGTGGGAAGGCGTGCCCTTCACGCCAAAATCCGTGAAGCTGGAAACCGATGAACAGATCGCCGCCCATGCGCGCGAAATCTATTTGCAAGCCGGCCGCAGCCATGCCAT
 A  E  P  V  W  E  G  V  P  F  T  P  K  S  V  K  L  E  T  D  E  Q  I  A  A  H  A  R  E  I  Y  L  Q  A  G  R  S  H  A  M
                                                                                                              BamHI
GCCTCCCGGCAACATCACCGCCATCACCCCGGATGAGCGCAAGGTGCTGACCGCCTGGTACGAAAGCGCGGTTTCCGGGGCCGGAAAAGCCGAAGGAAAGACTGAATGAGGATCCGGCTG
GCCTCCCGGCAACATCACCGCCATCACCCCGGATGAGCGCAAGGTGCTGACCGCCTGGTACGAAAGCGCGGTTTCCGGGGCCGGAAAAGCCGAAGGAAAGACTGAATGA-----------
 P  P  G  N  I  T  A  I  T  P  D  E  R  K  V  L  T  A  W  Y  E  S  A  V  S  G  A  G  K  A  E  G  K  T  E  * ----------
```

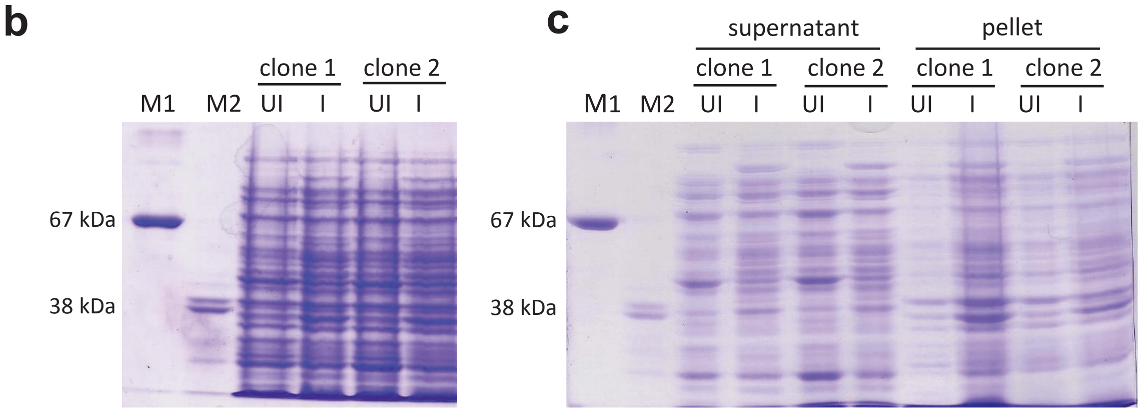

Supplementary Figure S11. **PuuD cloning and overexpression attempts**. (a) Alignment of the sequence inserted in the pET11b expression vector with the Atu2314 reference sequence. The pET11b ribosome binding site (rbs) and the NdeI and BamHI restriction sites are indicated; the synonymous ggT/ggC mutation is boxed. (b) SDS-PAGE of proteins from total lysate and (c) soluble and insoluble fractions of uninduced (UI) and induced (I) BL21 cells showing lack of overexpression of the recombinant PuuD protein (expected size ~48 kDa).

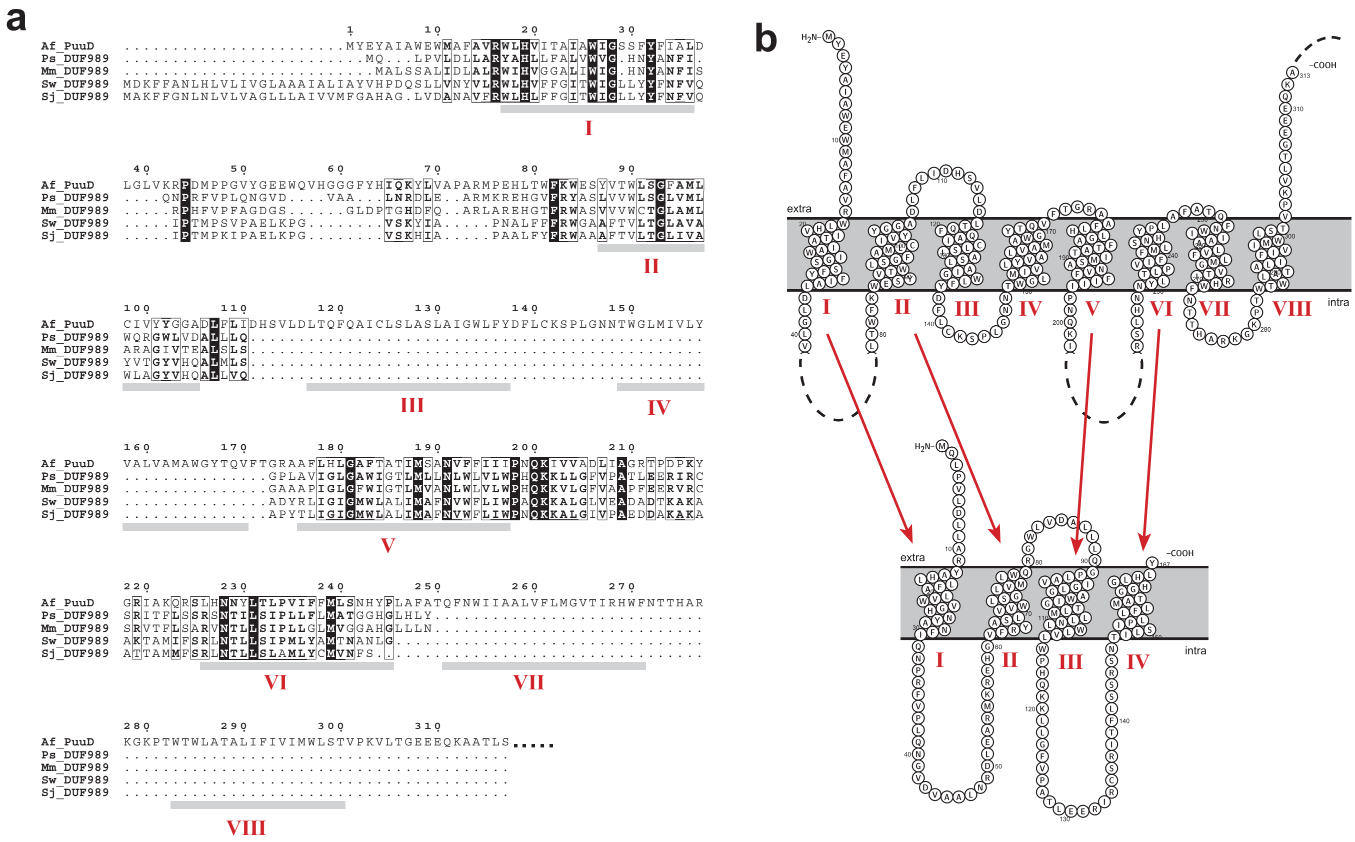

Supplementary Figure S12. **Comparison of PuuD with the short DUF989 variant**. (a) Multiple alignment of Af_PuuD with representative sequences of the short DUF989 variant from *Sphingobium japonicum* (Sj), *Pseudomonas stutzeri* (Ps), *Sphingomonas wittichii* (Sw), and *Magnetospirillum magnetotacticum* (Mm). The position of TM helices in AfPuuD is indicated by gray bars. (b) Comparison of the topological diagrams of Af_PuuD (upper) and Ps_DUF989 (lower) highlighting the correspondence between TM helices.

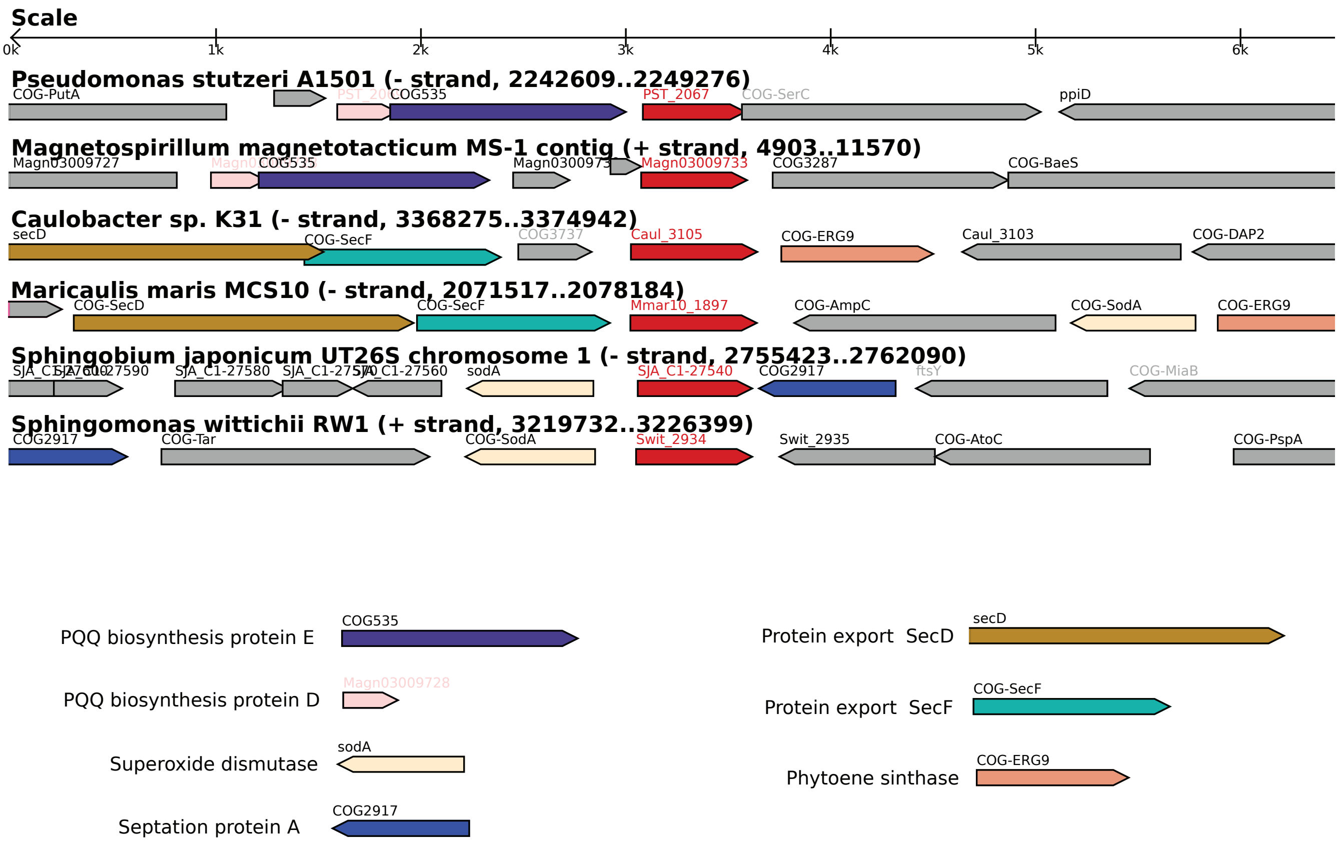

Supplementary Figure S13. **Neighborhood of genes encoding the short DUF989 variant**. A region of 6.5 Kb around genes encoding the DUF989 short variant (red) is shown for selected genomes. Homologous genes identified in the same region in different genomes are shown in color as indicated by the figure legend. Gene neighborhood was obtained with the Microbesonline web server (http://www.microbesonline.org).

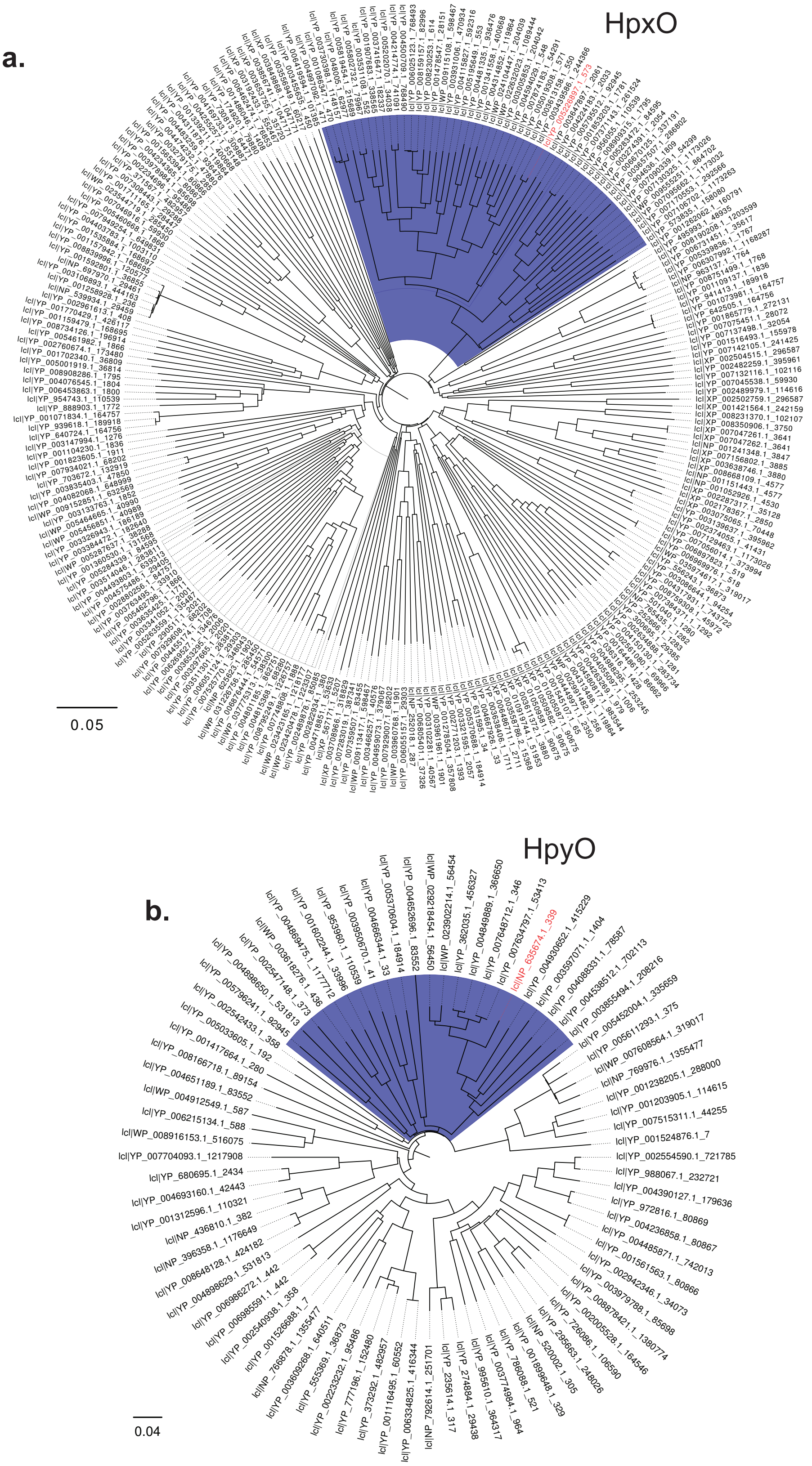

Supplementary Figure S13. **Selection of HpxO and HpyO proteins in the family phylogeny**. The trees represent neighbor joining phylogenies of clusters obtained by MCL analysis of sequences identified by homology with the Pfam domains (a) FAD_binding_3 (HpxO) and (b) NAD_binding_9 (HpyO). Experimentally characterized HpxO and HpyO proteins are coloured red. Nodes representing genes considered to be isofunctional are shaded in blue.
